# Primary Stenting of the Superficial Femoral Artery in Patients with Intermittent Claudication Has Durable Effects on Health-Related Quality of Life at 24 Months: Results of a Randomized Controlled Trial

**DOI:** 10.1007/s00270-018-1925-0

**Published:** 2018-03-08

**Authors:** Hans I. V. Lindgren, Peter Qvarfordt, Stefan Bergman, Anders Gottsäter, The Swedish Endovascular Claudication Stenting Trialists, I. Jansson, E. Litterfeldt, Hans I. V. Lindgren, Peter Qvarfordt, T. Fransson, A. Öjersjö, A. Hilbertson, T. Röjlar, Anders Gottsäter, G. Gruber, T. Hörer, T. Larzon, T. Jonasson, C. Strandberg, P. Andersson, Stefan Bergman, L. Lundell, A. Svensson, M. Warvsten

**Affiliations:** 1 Department of Clinical Sciences, Faculty of Medicine, Lund University, Lund, Sweden; 2 Department of Interventional Radiology and Surgery, Helsingborg Hospital, 251 87 Helsingborg, Sweden; 3 Vascular Centre, Skåne University Hospital, 205 02 Malmö, Sweden; 4 Primary Health Care Unit, Department of Public Health and Community Medicine, Institute of Medicine, The Sahlgrenska Academy, University of Gothenburg, Gothenburg, Sweden; 5 Spenshult Research and Development Centre, Halmstad, Sweden

**Keywords:** Peripheral arterial disease, Intermittent claudication, Primary stenting, Health-related quality of life, Superficial femoral artery

## Abstract

**Background:**

Intermittent claudication (IC) is commonly caused by lesions in the superficial femoral artery (SFA), yet invasive treatment is still controversial and longer term patient-reported outcomes are lacking. This prospective randomized trial assessed the 24-month impact of primary stenting with nitinol self-expanding stents compared to best medical treatment (BMT) alone in patients with stable IC due to SFA disease on health-related quality of life (HRQoL).

**Methods:**

One hundred patients with stable IC due to SFA disease treated with BMT were randomized to either stent (*n* = 48) or control (*n* = 52) group. HRQoL assessed by Short Form 36 Health Survey (SF-36) and EuroQoL 5-dimensions (EQ5D) 24 months after treatment were primary outcome measures. Walking Impairment Questionnaire, ankle–brachial index (ABI), and walking distance were secondary outcomes.

**Results:**

Significantly better SF-36 Physical Component Summary (*P* = 0.024) and physical domain scores such as Physical Function (*P* = 0.012), Bodily Pain (*P* = 0.002), General Health (*P* = 0.037), and EQ5D (*P* = 0.010) were reported in intergroup comparison between the stent and the control group. Both ABI (from 0.58 ± 0.11 to 0.85 ± 0.18; *P* < 0.001 in the stent group and from 0.63 ± 0.17 to 0.69 ± 0.18; *P* = 0.036 in the control group) and walking distance (from 170 ± 90 m to 616 ± 375 m; *P* < 0.001 in the stent group and from 209 ± 111 m to 331 ± 304 m; *P* = 0.006 in the control group) improved significantly in intragroup comparisons.

**Conclusions:**

In patients with IC caused by lesions in the SFA, primary stenting compared to BMT alone was associated with significant improvements in HRQoL, ABI, and walking distance durable up to 24 months of follow-up.

*Clinical Trial Registration* http://www.clinicaltrials.gov. Unique Identifier: NCT01230229

## Introduction

Peripheral arterial disease (PAD) is a global problem and intermittent claudication (IC) affects 20–40 million individuals worldwide. It is more common in high-income countries [1] with a prevalence of 7% in the Swedish population aged 60–90 years [2]. Treatment aims at cardiovascular risk factor modification through best medical treatment (BMT) and improvement in health-related quality of life (HRQoL) by increasing walking capacity. BMT with or without supervised exercise training (SET) is the generally accepted first-line therapy [3, 4], but revascularization is nowadays frequently performed, partly due to development of endovascular treatment [5, 6].

Results of invasive treatment of IC patients due to suprainguinal disease are excellent [7], but invasive treatment of the most common form of IC, i.e., infrainguinal IC caused by lesions in the superficial femoral artery (SFA), is hampered by high restenosis rates [8] and considered controversial according to international guidelines [3, 4]. Invasive treatment for IC should provide the best HRQoL for the patient with minimal complications, and PTA with selective stent implantation or primary stent implantation has significantly better results than PTA alone in this regard [9]. The use of primary SFA stenting has increased in recent years as primary patency and safety of the procedure have both improved [10].

Few trials include IC patients with lesions limited to the SFA [11]; however, long-term patient-reported outcomes including HRQoL are lacking in this group of patients.

The primary aim of this study was to assess HRQoL in patients with stable IC due to SFA disease 24 months after randomization to either SFA stenting in addition to BMT or BMT alone. A 12-month interim analysis of the study has previously been reported [12].

## Materials and Methods

In a 24-month open-label, randomized, controlled, two-armed study conducted at 7 Swedish hospitals; (Eskilstuna, Helsingborg University Hospital, Kalmar, Kristianstad, Örebro University Hospital, Skåne University Hospital Malmö, and Växjö) IC patients already on BMT were randomized on a 1:1 basis to primary SFA stenting versus control with BMT alone. The study design has previously been reported in detail [12]. The trial is registered in the ClinicalTrials.gov database (Identifier: NCT01230229).

Adult patients with stable (i.e., > 6 months) IC (Fontaine II b, Rutherford II–III) [13], with absolute walking capacity < 500 m measured by a standardized constant treadmill test (speed 3 km/h, without incline), caused by SFA stenosis or occlusion were included. The target treatment segment was the full length of the SFA, TASC II a–c [3] measured on preinclusion magnetic resonance tomography angiography (MRA) or computed tomography angiography (CTA). A patent popliteal and at least one patent non-stenotic tibial runoff artery were required for study inclusion. Patients with hemorrhagic stroke within the past 3 months, aneurysm in the SFA or popliteal artery, previously implanted stent(s) at the same site or poor aortoiliac or common femoral inflow were excluded from the study. Invasive correction of reduced inflow 3 months prior to evaluation of eligibility was allowed to enable randomization, however.

Other exclusion criteria were critical limb ischemia (CLI, Fontaine III and IV) [13], life expectancy < 24 months, and previous enrollment in this or other clinical trial.

As described in detail in the interim analysis [12], at telephone call from the treating physician patients were randomized on a 1:1 basis at the Spenshult Hospital research unit (located apart from any of the including study sites) by sealed envelopes containing allocations to the two study groups. Stratification was performed with regard to lesion length of less (short lesions) or more (long lesions) than 90 mm. Data were reported to research units at Helsingborg University Hospital and Spenshult Hospital. Source data verification was conducted by project monitors at Helsingborg University Hospital research unit. The treatment protocol remained unchanged throughout the study.

### Interventions

Subjects in both groups received antiplatelet (aspirin 75 mg/d or clopidogrel 75 mg/d), lipid lowering, and antihypertensive drugs with treatment targets according to Swedish guidelines current at study start: total cholesterol 4.5 mmol/l, LDL-cholesterol 2.5 mmol/l, and blood pressure (BP) 140/90 mmHg in patients without diabetes mellitus (130/80 mm Hg in patients with diabetes mellitus) [14], in addition to instructions about regular exercise. Follow-up visits were performed at 1, 6, 12, and 24 months. In the absence of a standardized program for SET in Sweden, patients received a pedometer and were further encouraged to exercise when readouts were recorded at all follow-up visits. Smokers were actively advised to quit, with help from a smoke cessation unit if needed.

For patients in the stent treatment group, modern nitinol bare metal stents (BMS) designed for the SFA with 1 mm larger diameter than the reference vessel were selected and deployed by the treating physician. Stent length was chosen to cover the lesion with one stent if possible. If more than one stent was required, overlap (between 0.5 and 1 cm) was accepted. Stents were placed to allow at least 5-mm lesion-free vessel at both ends. A calibrated angiogram compared pre- and post-implant minimum lumen diameters and measured non-diseased artery diameters, where after residual percent stenosis was calculated. Before crossing the lesion, an i.v. heparin bolus of 5000 units was administered. Further heparin was given with guidance from Active Clotting Time (ACT)—target value 250 s. Stented patients were treated with aspirin 75 mg daily, which during the first 12 weeks after stenting was combined with clopidogrel 75 mg daily (without loading dose). In stented patients with ongoing anticoagulant treatment, aspirin was added for 3 months after endovascular treatment.

All patients were evaluated by a vascular surgeon, vascular physician, or interventional radiologist, and research nurse at a hospital outpatient visit 1, 6, 12, and 24 months after inclusion.

### Primary Outcome Measure

Change in HRQoL between baseline and 24 months using the Short Form 36 Health Survey (SF-36, rating HRQoL 0–100 from worst to best in 8 domains) [15] and EuroQoL 5-dimensions (EQ 5D, rating HRQoL states 0–1 from worst to best) [16] was primary outcome measures. SF-36 consists of 8 domains (Physical Function [PF], Role Physical [RP], Bodily Pain [BP], General Health [GH], Vitality [VT], Social Function [SF], Role Emotional [RE] and Mental Health [MH]). Two linear combinations were computed from these 8 domains: a Physical Component Summary (PCS), and a Mental Component Summary (MCS).

### Secondary Outcome Measures

Change in Walking Impairment Questionnaire (WIQ, rating 0–100 from worst to best) [17], ABI, and absolute walking distance was measured by a standardized treadmill test (speed 3 km/h, without incline, maximum duration 20 min or 1000 m) between baseline and 24 months.

### Additional Measurements

Procedure-related adverse events, restenosis measured by duplex ultrasound, reinterventions in the stent group, cardiovascular events, and crossovers between groups were prospectively recorded at the study sites and reported to the research unit at Helsingborg Hospital.

### Statistical Analysis

The sample size was determined to study a clinically relevant difference between the two groups with regard to the primary outcome variable. With a significance level of 5% and with 50 patients in each group, a clinically important difference of 10 points in SF-36 [18, 19] could be detected with a power of at least 80%.

Comparisons were performed at 5% significance level based on two-sided tests. Results are presented with 95% CI and *P* values. Differences in continuous variables between groups were analyzed using the intention-to-treat (ITT) principle. *T* tests and paired *t* tests were conducted, and mean difference and the correspondent two-sided 95% CI are reported.

### Ethical Issues

The study was performed in accordance with the spirit of the Declaration of Helsinki and in agreement with the guidelines for conducting a clinical investigation as outlined in the ISO 14-155. Written informed consent was obtained from all patients. The study was approved by the Medical Ethics Committee of Lund University (Dnr 2009/478).

## Results

### Participant Flow

**Fig. 1:**
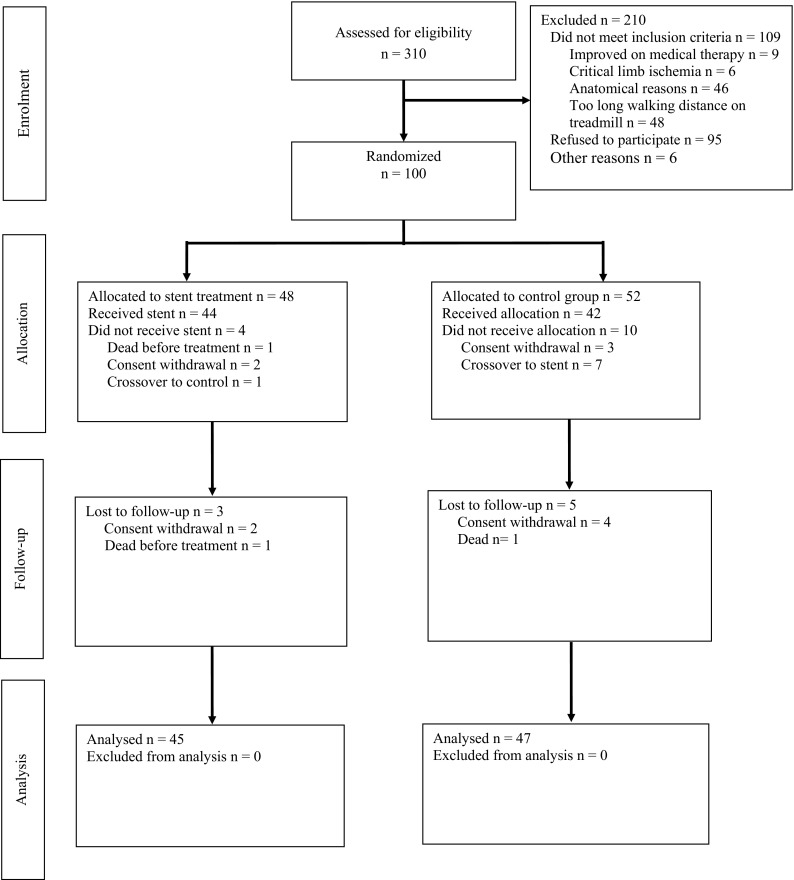
CONSORT [40] diagram for trial

Between 2010 and 2015, 310 patients were screened at the 7 study sites, whereof 100 patients were randomized. Forty-eight patients (22 with long lesions and 26 with short lesions) were randomized to the stent group, and 52 patients (26 with long lesions and 26 with short lesions) to the control group (Fig. 1). Results are reported from the 92/100 patients in whom 24-month follow-up data could be analyzed. (Four patients in the control group and two in the stent group withdrew their consent. One patient in the stent group died before stent treatment, and one patient in the control group died from heart failure after 15 months).

### Baseline Data

Groups were well matched at baseline concerning background variables such as age, sex, smoking habits, BP, LDL-cholesterol, duration of IC (Table 1), lesion characteristics (except lesions length that were longer in the stent group), and HRQoL at baseline did not differ between groups (Table 2).

**Table 1:** Baseline demographic data and lesion characteristics by treatment group. Mean (SD) or *n* (%)

	Stent	Control	*P* value
Age (years)	71.3 (5.3)	69.8 (5.8)	0.184
Sex			0.540
Male	23	23	
Female	28	21	
ABI	0.58 (.125)	0.63 (.171)	0.129
Walking distance (m)	178 (87.3)	210 (107.4)	0.114
Duration of IC (months)	30 (29.3)	41 (48.3)	0.179
Smoking			0.125
Yes (*n*)	7	11	
Former (*n*)	27	32	
Never (*n*)	12	5	
LDL (mmol/l)	2.75 (1.1)	2.55 (.9)	0.374
B-glucose (mmol/l)	7.0 (2.8)	6.3 (2.1)	0.207
Systolic BP (mmHg)	155 (21.7)	150 (20.7)	0.282
Diastolic BP (mmHg)	80 (11.3)	79 (8.8)	0.831
S-creatinine (µmol/l)	84 (24.5)	82 (21.9)	0.653
Lesion length (mm)	145 (91)	103 (97)	0.021
Occlusion (*n*)	31	36	0.654
Stenosis (*n*)	14	13	NS
Degree of stenosis (%)	81.7 (16.3)	91.5 (3.1)	0.065
No crural vessels (*n*)	2.5 (.6)	2.3(.7)	0.219

B = blood, P = plasma, S = serum, BP = blood pressure, IC = intermittent claudication, LDL = low-density protein, ABI = ankle–brachial index

**Table 2:** Baseline and 24-month levels of primary and secondary outcome variables in patients with intermittent claudication (IC) with primary stenting (stent, *n* = 45) or best medical treatment only (control, *n* = 47). Mean (SD)

	Baseline			24 months		
	Stent	Control	*P* value	Stent	Control	*P* value
PF	43 (17)	43 (17)	0.972	60 (22)	48 (19)	0.012
RP	41 (39)	43 (41)	0.731	47 (42)	43 (41)	0.748
BP	40 (17)	38 (17)	0.734	56 (25)	40 (20)	0.002
GH	54 (17)	53 (20)	0.867	58 (22)	48 (19)	0.037
VT	49 (22)	50 (23)	0.686	57 (24)	48 (19)	0.059
SF	74 (23)	72 (30)	0.648	80 (19)	71 (27)	0.090
RE	56 (44)	58 (44)	0.801	62 (44)	58 (44)	0.742
MH	72 (21)	72 (24)	0.861	75 (22)	68 (21)	0.169
PCS	31 (8)	31 (7)	0.912	38 (11)	33 (8)	0.024
MCS	48 (12)	49 (14)	0.646	48 (12)	46 (13)	0.471
EQ5D	0.56 (0.27)	0.46 (0.31)	0.121	0.65 (0.26)	0.48 (0.32)	0.010
WIQ	40 (18)	35 (18)	0.175	56 (26)	41 (22)	0.007
ABI	0.6 (0.1)	0.6 (0.2)	0.129	0.9 (0.2)	0.7 (0.2)	< 0.001
WD	171 (90)	209 (106)	0.114	615 (375)	331 (304)	< 0.001

Short Form 36 Health Survey (SF-36, rating HRQoL 0–100 from worst to best in eight domains (Physical Function [PF], Role Physical [RP], Bodily Pain [BP], General Health [GH], Vitality [VT], Social Function [SF], Role Emotional [RE], Mental Health [MH]), Physical Component Summary (PCS), Mental Component Summary (MCS) [15], EuroQoL 5-dimensions (EQ 5D, rating health-related quality of life states 0–1 from worst to best) [16], and Walking Impairment Questionnaire (WIQ, rating 0–100 from worst to best) [17]

Ankle–brachial index (ABI), walking distance (WD)

### Primary Outcome Measure at 24 Months

Significantly better SF-36 PCS score (*P* = 0.024) and physical domain scores PF (*P* = 0.012), BP (*P* = 0.002), GH (*P* = 0.037), and EQ 5D (*P* = 0.010) were reported in intergroup comparison between the stent and the control group (Table 2).

**Fig. 2:**
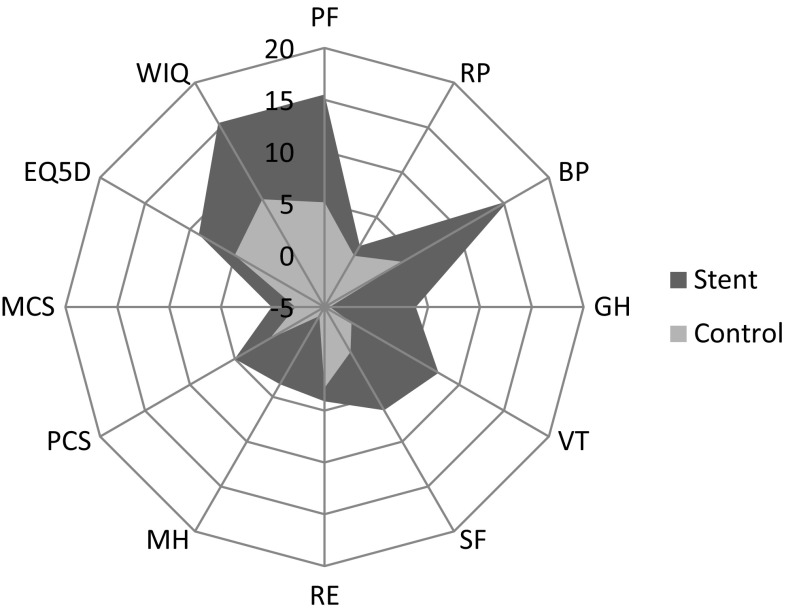
Spider plot of 24-month changes in primary and secondary outcome measures in patients with intermittent claudication (IC) with primary stenting (stent) or only best medical treatment (control). Short Form 36 Health Survey (SF-36, rating HRQoL 0–100 from worst to best in eight domains (Physical Function [PF], Role Physical [RP], Bodily Pain [BP], General Health [GH], Vitality [VT], Social Function [SF], Role Emotional [RE] Mental Health [MH]), Physical Component Summary (PCS), and a Mental Component Summary (MCS) [15], EuroQoL 5-dimensions (EQ5D, rating health related quality of life states 0–1 from worst to best, shown as % of 1) [15], and Walking Impairment Questionnaire (WIQ, rating 0–100 from worst to best) [15]. Ankle brachial distance (ABI). Walking distance (WD)

In intragroup comparisons, the SF-36 PCS score (*P* = 0.002) and SF-36 domain scores such as PF (*P* < 0.001), BP (*P* < 0.001), and VT (*P* = 0.018) improved significantly in the stent group, whereas no primary outcome measure improved in the control group (Table 3; Fig. 2).

**Table 3:** Twenty-four month changes in primary and secondary outcome measures in patients with intermittent claudication (IC) with primary stenting (stent) or only best medical treatment (control)

	Stent group			Control group		Stent group versus Control group	
	Mean change (95% CI)	*P*		Mean change (95% CI)	*P*	Mean difference of improvement (95% CI)	*P*
PF	15.5 (9.3–21.8)	<0.001		5.1 (− 1.5–10.4)	0.057	10.4. (2.3–18.4)	0.012
RP	1.8 (− 7.3–10.8)	0.697		0.7 (− 12.9–14.3)	0.918	1.1 (− 15.0–17.2)	0.896
BP	15.4 (7.6–23.1)	<0.001		3.7 (− 1.9–9.3)	0.184	11.6 (2.2–21.1)	0.016
GH	3.8 (− 1.6–9.3)	0.160		− 4.5 (− 9.4–0.5)	0.078	8.3 (1.1–15.6)	0.025
VT	7.7 (1.4–14.0)	0.018		− 2.0 (− 8.3–4.4)	0.541	9.7 (0.8–18.6)	0.033
SF	6.5 (− 0.5–13.6)	0.068		0.0 (− 5.7–5.7)	1.0	6.6 (− 2.4–15.5)	0.147
RE	4.1 (− 9.1–17.5)	0.529		2.8 (− 10.3–16.0)	0.661	1.3 (− 17.0–19.6)	0.887
MH	3.6 (− 3.1–10.3)	0.286		− 4.0 (− 10.3–2.2)	0.200	7.6 (− 1.4–16.6)	0.097
PCS	5.0 (2.0–8.0)	0.002		1.1 (− 1.3–3.6)	0.355	3.9 (0.1–7.7)	0.047
MCS	0.1 (− 3.9–4.0)	0.985		− 2.2 (− 5.3–0.8)	0.150	2.3 (− 2.6–7.3)	0.359
EQ5D	0.09 (− 0.01–0.20)	0.071		0.05 (− 0.03–0.13)	0.241	0.04 (− 0.01–0.17)	0.527
WIQ	15.5 (7.2–23.9)	0.001		7.0 (0.1–14.0)	0..047	8.4 (− 2.2–19.3)	0.120
ABI	0.28 (0.20–0.35)	<0.001		0.05 (0.0–0.1)	0.036	0.2 (0.12–0.29)	<0.001
WD	614 (491–738)	<0.001		337 (242–431)	<0.001	305 (156–456)	<0.001

Short Form 36 Health Survey (SF-36, rating HRQoL 0–100 from worst to best in eight domains (Physical Function [PF], Role Physical [RP], Bodily Pain [BP], General Health [GH], Vitality [VT], Social Function [SF], Role Emotional [RE] Mental Health [MH]), Physical Component Summary (PCS), Mental Component Summary (MCS) [15], EuroQoL 5-dimensions (EQ5D, rating health-related quality of life states 0–1 from worst to best) [16], and Walking Impairment Questionnaire (WIQ, rating 0–100 from worst to best) [17], ankle–brachial index (ABI), walking distance (WD)

### Secondary Outcome Measures at 24 Months

In intergroup comparison, significantly better WIQ score was reported in the stent group (*P* = 0.007; Table 2). In intragroup comparison, WIQ improved significantly in both the stent group (*P* = 0.001) and the control group (*P* = 0.047; Table 3; Fig. 2).

Both ABI (0.19; *P* < 0.001) and walking distance (284 m; *P* < 0.001) were significantly better in the stent group in intergroup comparison at 24 months (Table 2).

Both ABI (from 0.58 ± 0.11 to 0.85 ± 0.18; *P* < 0.001 in the stent group, and from 0.63 ± 0.17 to 0.69 ± 0.18; *P* = 0.036 in the control group) and walking distance (from 170 ± 90 m to 616 ± 375 m; *P* < 0.001 in the stent group, and from 209 ± 111 m to 331 ± 304 m; *P* = 0.006 in the control group) improved significantly in intragroup comparisons (Table 3; Fig. 2).

Seventeen patients in the stent group reached the maximum walking distance of 1000 m, whereas only 6 patients in the control group reached this limit (*P* < 0.001).

### Additional Measurements at 24 Months

All lesions were successfully recanalized and treated without bailout procedures, or complications causing prolonged hospitalization.

Duplex ultrasound in the stent group revealed four stent occlusions, five significant in stent restenoses, and one new stenosis above the stented SFA segment.

Nine target lesion revascularizations and one target vessel revascularization (due to a new stenosis above the stented lesion) were successfully performed in 9 cases within 24 months. One patient was treated with a femoro-popliteal bypass due to stent occlusion at 10 months, hereafter deteriorated and was amputated below the knee at 18 months. One patient randomized to stenting died already before treatment, and one patient in the control group died of congestive heart failure after 15 months (mortality rate 2%). No other amputations or deaths occurred.

Seven patients in the control group received stent treatment after 3, 7, 11, 12, 13, 15, and 16 months due to deterioration to either disabling IC or CLI.

Other severe adverse events (SAE) leading to hospitalization during the 24-month follow-up were as follows: 7 hospitalizations for atrial fibrillation (5 in the stent group and 2 in the control group), 5 for myocardial infarction (3 in the stent group and 2 in the control group), 2 for ischemic stroke (in the stent group), and 1 for gastrointestinal bleeding (during dual antiplatelet treatment in the stent group). Ten other SAE not related to the cardiovascular system occurred.

Systolic BP was unchanged in both the stent (151 ± 21 mmHg and 146 ± 20 mmHg; *P* = 0.10) and the control (149 ± 21 mmHg and 146 ± 20 mmHg; *P* = 0.19) group.

LDL-cholesterol levels were unchanged in both the stent (2.6 ± 1.0 mmol/l and 2.6 ± 1.1 mmol/l; *P* = 0.726) and the control (2.5 ± 0.8 mmol/l to 2.4 ± 0.8 mmol/l; *P* = 0.069) group.

## Discussion

This randomized controlled study demonstrates for the first time significant durable improvement in HRQoL after primary stenting of SFA lesions combined with BMT, as compared to BMT alone.

Furthermore, statistically significant improvement measured by the disease-specific questionnaire WIQ also occurred in the stent group. Improvements in other secondary endpoints ABI and walking distance, on the other hand, were recorded in both groups but were of much larger magnitude and clinical relevance in the stent group.

The reported improvement in HRQoL in the stent group is mainly attributed to substantial improvement in physical function and reduction in pain (Tables 2, 3; Fig. 2) which was not seen in the control group. We have previously reported on the prevalence of pain and importance of pain reduction in relation to treatment of PAD [20], and our results confirm the previously reported [21] great importance of pain-free walking distance for HRQoL.

Benefit from invasive treatment of IC has also been reported in previous studies [21–23], but it should be noted that these have evaluated different interventional techniques in heterogeneous patient materials with mixed supra- and infrainguinal lesion. Whereas other attempts to exclusively study patients with IC caused by SFA disease have failed due to difficulties in recruitment of patients [24], this study for the first time demonstrate durable benefit of invasive treatment in this commonly occurring type of IC patients.

In a previously reported interim analysis [12], a possible explanation for the relatively limited effects of continued BMT alone on HRQoL was given; stable claudication and 6 months of medical treatment were prerequisites for randomization.

The quality of BMT is important and might influence the long-term prognosis and reduce the risk of major cardiovascular events in patients with PAD [3, 25]. In spite of the fact that risk factor control was acceptable in this study without differences between the study groups concerning BP, LDL-cholesterol, or smoking habits during 24 months of follow-up, both the fact that 15 patients were hospitalized for cardiovascular complications not related to the lower extremities, and the death from congestive heart failure occurring in the control group highlight the importance of atherosclerotic comorbidities in this group of patients.

Conservative treatment, e.g., SET and BMT, of risk factors is recommended in infrainguinal IC [3, 4]. Positive effects of BMT have been documented up to 18 months [26], and improvement in walking distance by SET has been reported up to 24 months [27].

As SET programs are not available in the Swedish health care system, the study protocol was constructed to reflect the “real-world” situation at 7 different hospitals. This is of course a scientific study limitation, but on the other hand constitutes also a pragmatic solution which might increase the generalizability of the study. To simulate SET, patients were actively encouraged to regular walking promoted by offering each patient a pedometer and a protocol for registration of walking distances. However, the suboptimal treatment compliance in the BMT group of 81% (3 consent withdrawals and 7 crossovers to stenting due to deterioration to either disabling IC or CLI) might raise a question mark on the efficacy of this regime. In a previous study, greater improvement in walking distances and HRQoL scores of additional endovascular treatment compared with SET alone up to 12 months was demonstrated [28]. It is interesting to note that the present study demonstrated that such additional effects on HRQoL of invasive treatment were durable even up to 24 months.

The SFA has a complicated movement pattern [29], causing more stent fractures and restenoses than in other stented vascular territories [30] more pronounced in longer SFA lesions requiring longer/overlapping stents [31–33]. The common need for reinterventions is well known, and in recent treatment guidelines [4] stent placement in infrainguinal lesions is still considered controversial and only recommended for lesions up to 25 cm. In this context, it must be taken into account that HRQoL was the primary outcome measure of this study comparing primary SFA stenting with BMT alone. The study was therefore not powered to investigate the primary patency and safety of primary stenting with BMS, but it is nevertheless interesting to note that our 24-month primary and secondary patency rates of 80 and 96%, respectively, are well in line with results previously reported in a meta-analysis of 11 prospective clinical trials of SFA stenting during 12-month follow-up [10]. It is also noteworthy that results on HRQoL in this study were durable at 24 months in spite of the relatively long lesions and stented SFA segments.

A comparison of drug-eluting stents (DES) with PTA with bailout stenting [34] on SFA lesions showed better patency and event free survival with DES than with PTA. That study did not report on HRQoL and did not directly compare DES with BMS, but the reported 24-month primary patency of 74.8% of this trial [35] and of 83.5% of another recent DES trial [36] is comparable with our results.

Studies with drug-eluting balloons (DEB) have also shown superior results compared to PTA [37–39], but to the best of our knowledge no study has yet compared DEB with primary stenting or the impact of any drug-eluting technology compared to BMT alone on HRQoL.

### Limitations

Our strict inclusion and exclusion criteria resulted in a well-characterized patient material with the most common type of IC caused by lesions in the SFA. This can of course also be considered as a study limitation, however, as the results are possible to generalize only to patients with stable IC meeting our narrow eligibility criteria. The large number of screening failures due to the strict inclusion and exclusion criteria might have created a bias in patient selection, but this is unlikely as baseline characteristics were comparable in both groups.

Treatment was not blinded for patients as “sham” stenting was not considered ethical acceptable, and this might have, due to a potential placebo effect, introduced a bias in HRQoL reporting.

Possible bias due to a difference between the groups in medical treatment with stented patients treated with dual antiplatelet therapy during the first 12 weeks after stenting cannot be excluded.

The high number of comparisons made in the study increases the risk of rejecting a true null hypothesis, and caution is necessary when interpreting the actual significance of *P* values.

## Conclusion

In patients with IC caused by lesions in the SFA, primary stenting compared to BMT alone was associated with significant improvements in HRQoL, ABI, and walking distance durable up to 24 months of follow-up.
